# Application of new statistical distribution approaches for environmental mixture risk assessment: A case study

**DOI:** 10.1016/j.scitotenv.2019.07.316

**Published:** 2019-11-25

**Authors:** Aude Kienzler, Stephanie Bopp, Marlies Halder, Michelle Embry, Andrew Worth

**Affiliations:** a European Commission, Joint Research Centre, Via E. Fermi, 2749, 21027 Ispra, VA, Italy; b Health and Environmental Science Institute, 740 15th Street NW, Suite 600, Washington, DC 20005, USA

**Keywords:** AF, assessment factor, CA, concentration addition, CTD-TC, chemical toxicity distribution-threshold concentration, CY, Cyprus, ECHA, European Chemicals Agency, ecoTTC, ecotoxicological Threshold of Toxicological Concern, EFSA, European Food Safety Authority, FR, France, MEC, measured exposure concentration, MOA, mode of action, MRA, mixture risk assessment, MLT, Malta, PFOA, perfluorooctanoic acid, PFOS, perfluorooctanesulfonic acid, PNEC, predicted no-effect concentration, QSAR, quantitative structure–activity relationship, REACH, Registration, Evaluation, Authorisation and Restriction of Chemicals, RQ_i_, risk quotient for the individual substance, RQ_mix_, risk quotient for a mixture, STU, sum of toxic units, SW, Sweden, TTC, Threshold of Toxicological Concern, TU, toxic unit, Ecotoxicological Threshold of Toxicological Concern, Aquatic toxicity, Combined exposure, Mode of action

## Abstract

**Objectives:**

There is growing evidence that single substances present below their individual thresholds of effect may still contribute to combined effects. In component-based mixture risk assessment (MRA), the risks can be addressed using information on the mixture components. This is, however, often hampered by limited availability of ecotoxicity data. Here, the possible use of ecotoxicological threshold concentrations of no concern (i.e. 5th percentile of statistical distribution of ecotoxicological values) is investigated to fill data gaps in MRA.

**Methods:**

For chemicals without available aquatic toxicity data, ecotoxicological threshold concentrations of no concern have been derived from Predicted No Effect Concentration (PNEC) distributions and from chemical toxicity distributions, using the EnviroTox tool, with and without considering the chemical mode of action. For exposure, chemical monitoring data from European rivers have been used to illustrate four realistic co-exposure scenarios. Based on those monitoring data and available ecotoxicity data or threshold concentrations when no data were available, Risk Quotients for individual chemicals were calculated, to then derive a mixture Risk Quotient (RQmix).

**Results:**

A risk was identified in two of the four scenarios. Threshold concentrations contribute from 24 to 95% of the whole RQmix; thus they have a large impact on the predicted mixture risk. Therefore they could only be used for data gap filling for a limited number of chemicals in the mixture. The use of mode of action information to derive more specific threshold values could be a helpful refinement in some cases.

## Highlights

• The EnviroTox database and tool is a powerful tool to derive environmental threshold concentrations.
• The ecoTTC approach can be used for data gap filling.
• For mixture assessment, it can be used for a limited number of chemicals only.
• In some case the Mode of Action can be used to refine the surrogate value.

## Graphical abstract

**Figure:**
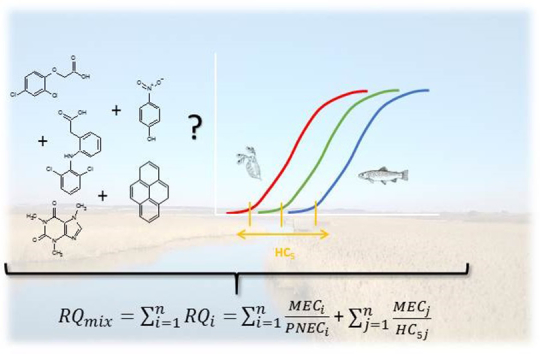

## 1 Introduction

Wildlife species are exposed to a large number of different combinations of chemicals entering the aquatic ecosystem via many sources. Although current risk assessment practice focuses on individual chemicals and their “no-effect” concentrations, there is evidence that single substances present below their individual thresholds of effect can still be of concern and contribute to combined effects (Kortenkamp et al., 2009; Silva et al., 2002), which is of particular importance given that chemicals are typically assessed separately within individual regulatory frameworks (Evans et al., 2016).

Since in many cases no data are available on ecotoxicological effects of a mixture or an environmental sample as a whole, it is common practice to predict combined effects and risks based on information on the mixture components. Various calculation approaches are used to predict mixture toxicity, most often based on the concentration addition (CA) concept (Kienzler et al., 2014), which assumes that the combined risk can be predicted from the sum of the potency corrected concentrations. CA is assumed to be applicable to combinations of chemicals with a similar mode of action (MOA) and to be an option for dissimilarly acting compounds as a first conservative approach (EFSA Panel on Plant Protection Products and Their Residues, 2013).

A framework for environmental mixture risk assessment (MRA) has been suggested by Backhaus and Faust (2012), combining two approaches based on index calculation. However, although the calculation approaches are rather simple, they require the knowledge of the exposure concentration and the ecotoxicological reference value for each chemical in the mixture, which are not always available. Consequently, MRA is often hampered by limited data availability (Bopp et al., 2016, Bopp et al., 2018; Kienzler et al., 2016).

An approach has been recently suggested, the ecotoxicological Threshold of Toxicological Concern (ecoTTC) approach, similar to the Threshold of Toxicological Concern (TTC) approach for humans. The TTC approach for human safety or risk assessment aims at setting a *de minimis* value below which exposure is considered unlikely to be a concern, based upon structural characteristics of the candidate chemical and existing toxicity data for other substances in an identified database (Munro et al., 1996). Those threshold values are derived from cumulative distributions of experimental No Observed Effect Levels (NOELs) on cancer and non-cancer toxicity endpoints. The TTC approach was originally developed for substances present at low levels in the diet, and was subsequently evaluated in detail for use in food safety by the European Food Safety Authority (EFSA, 2012, EFSA, 2019a). It was further improved (EFSA/WHO, 2016) and expanded to the risk assessment of other types of substances, including substances present in consumer products and cosmetics (SCCS, SCHER and SCENIHR, 2012; SCCS NfG, 2018) (Yang et al., 2017), micropollutants, drug residues, pesticide metabolites (EFSA, 2016; Houeto et al., 2012; Laabs et al., 2015; Mons et al., 2013); and genotoxic impurities in human pharmaceuticals (EMEA, 2006).

Similarly, the ecoTTC would allow for the calculation of environmental threshold concentrations of “no concern” (Belanger et al., 2015). An online tool, EnviroTox, has recently been developed to derive ecotoxicological threshold concentrations, based on the underlying EnviroTox database. This database of >91,000 curated records for acute and chronic aquatic toxicity data covers >3900 unique CAS numbers and 1560 aquatic species. The dataset was developed from 12 original databases (Connors et al., 2019; HESI, 2018a, HESI, 2018b). In addition to the ecotoxicological data, the database also includes other chemical information such as physicochemical properties and MOA classification according to several MOA frameworks (Verhaar, U.S. Environmental Protection Agency Assessment Tool for Evaluating Risk, U.S. Environmental Protection Agency Toxicity Estimation Software Tool, Laboratory of Mathematical Chemistry OASIS). These frameworks have been previously compared and discussed (Kienzler et al., 2017; Kienzler et al., 2019). The information on the MOA can be used for grouping chemicals, which might help for MRA (Bopp et al., 2015) and is also important in ecotoxicological risk assessment as it might help to focus on the most sensitive species and/or trophic level. Two types of threshold concentrations can be derived based on two different types of statistical distributions. The first type of threshold concentration is based on predicted no-effect concentration (PNEC) distributions, i.e., ecotoxicological value of interest for a given chemical to which an assessment factor (AF) is applied. The choice of the AF for PNEC derivation depends on several factors, such as 1) the coverage of the underlying dataset in terms of acute and chronic data, as well as species and trophic levels; and 2) the geographical region and thus the regulatory framework considered. Once the PNEC distribution is built, the 5th percentile of the distribution gives the threshold concentration, called the ecoTTC value.

The second type of threshold concentration is based directly on the distribution of the ecotoxicological values without applying any AF. In this paper, this type of threshold concentration will be called the chemical toxicity distribution-threshold concentration (CTD-TC). In both cases, the threshold concentration is derived by calculating the fifth percentile of the distribution.

An international World Health Organization/International Programme on Chemical Safety working group proposed a tiered approach for human health MRA (Meek et al., 2011), which involves integrated and iterative consideration of exposure and hazard at all phases, with each tier being more refined (i.e., less conservative and more certain) than the previous one, but more laborious and data intensive. In this framework, the use of the human TTC is proposed for screening level Tier 0 hazard assessment (Boobis et al., 2011). Since environmental chemical MRA is often hampered by limited data availability, the use of such threshold concentrations for screening level MRA and for data gap filling in MRA is explored in this work, in order to better understand if and to which extent this approach can be helpful for environmental MRA. Therefore, this work aims at answering several questions such as whether and how the threshold concentrations could be used in MRA; where they could fit in the above mentioned environmental MRA framework; if the threshold concentrations could be used in a similar way to how it has been used for human health; and how the MOA grouping could influence the MRA outcome.

## 2 Material and methods

**Fig. 1:**
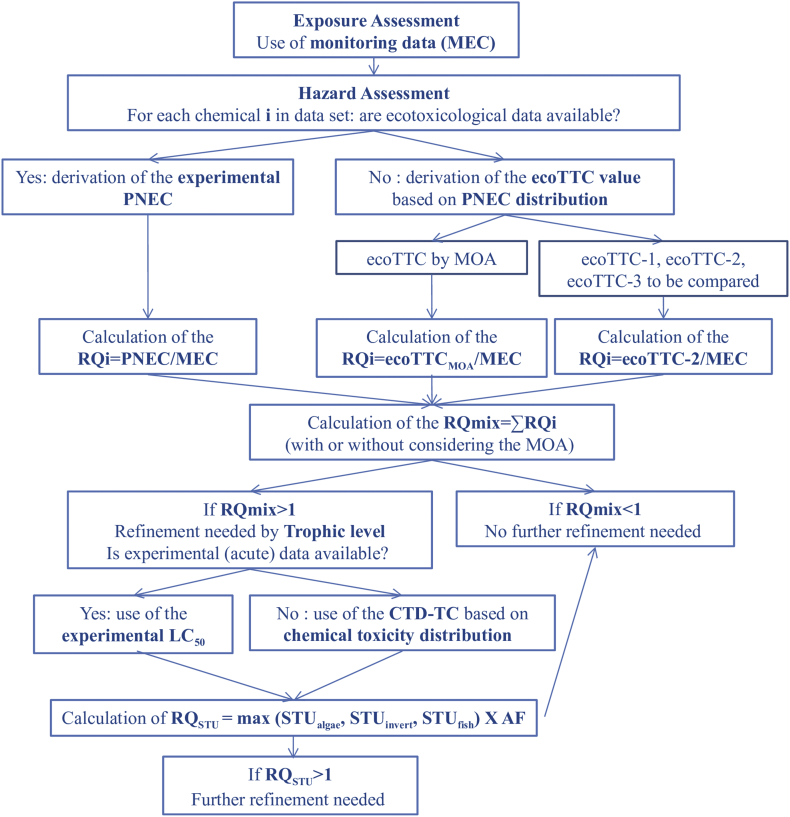
Methodology workflow used in the case study. *Abbreviations:* MEC, measured environmental concentration; ecoTTC, ecotoxicological Threshold Of Toxicological Concern; PNEC, predicted no-effect concentration; MOA, mode of action; RQi, risk quotient for the chemical i; RQmix, risk quotient for the mixture; CTD-TC, chemical toxicity distribution threshold concentration; RQ_STU,_ risk quotient for the mixture, based on the sum of the toxic unit; STU, sum of the toxic unit; AF, assessment factor.

The whole workflow is described in Fig. 1.

### 2.1 Exposure data

Chemical monitoring data from European rivers have been used in this case study (Loos et al., 2009). The complete dataset includes 35 chemicals of different types (pharmaceuticals, pesticides, perfluorooctanesulfonic acid [PFOS], perfluorooctanoic acid [PFOA], benzotriazoles, hormones) and >100 sampling locations across Europe. This monitoring program focused on polar organic persistent pollutants and aimed at obtaining an EU-wide overview on the occurrence of polar organic pollutants in European rivers. Therefore, it might miss some other type of pollutants. However, this dataset was chosen because it gives both realistic environmental concentrations and realistic co-exposure scenarios to a relevant number of chemicals, regulated across different regulatory frameworks. It has to be noted that not all 35 chemicals are always detected in all of the sampling sites. Among those 100 sampling locations, four exposure scenarios have been selected for this case study, corresponding to four different levels of pollution: Wied tal-Lunzjata (Malta [MLT]) and Gota Alv-Alelyckan (Sweden [SW]), low pollution level (between 150 and 310 ng/L of total chemical burden); Saone, Ile Barbe (France [FR]), medium pollution level (>850 ng/L of total chemical burden); and Garyllis/Lemesos (Cyprus [CY]), high pollution level (>27,000 ng/L). The complete list of chemicals and the four exposure scenarios are presented in Table S1. **Tert-Octylphenol (Tert-OP)** was the only chemical of the 35 monitored that was not found in any of those four locations. In this case study which aims at assessing the usefulness of threshold concentration for data gap filling, the chemicals that were not detected were not taken into consideration (i.e., their concentration were considered as 0).

### 2.2 Ecotoxicological reference value and PNEC derivation

#### 2.2.1 Based on experimental data available

Available ecotoxicological reference values have been gathered for the identified chemicals in each exposure scenario from regulatory sources if available, such as the European Chemicals Agency (ECHA) Database and the European Food Safety Authority (EFSA) OpenFoodTox database (ECHA, 2019; EFSA, 2019b). If no data were available from regulatory sources, they were searched in the literature or from existing databases, via the Organization for Economic Cooperation and Development QSAR Toolbox 4.0. The available ecotoxicological values are given in Table S2, as well as their sources, the AF applied, and the resulting PNEC. The AF for the PNEC derivation was chosen according to the ECHA (2008) guidance document. For some chemicals for which a regulatory PNEC was already calculated (i.e., in the ECHA Database), this PNEC was used.

#### 2.2.2 Data gap filling with threshold concentration

**Fig. 2:**
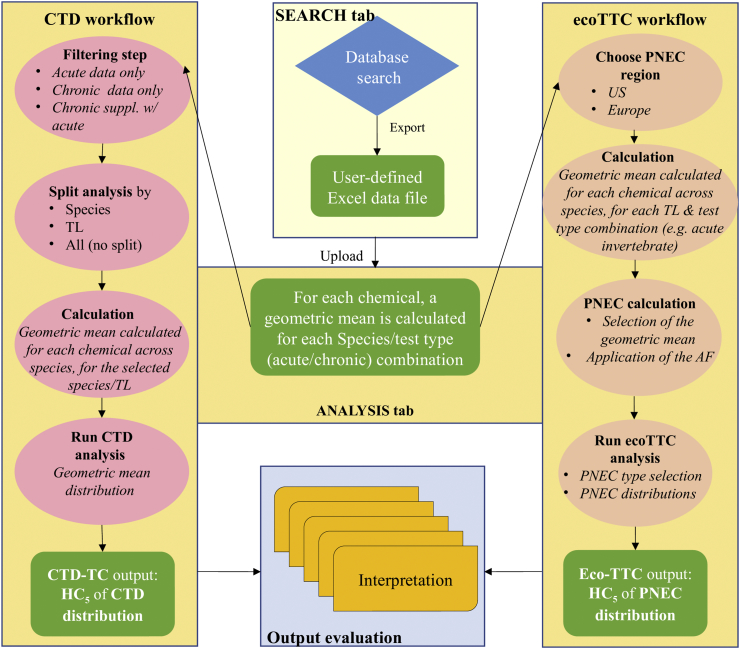
Dataset processing to obtain the two types of distribution (modified from (HESI, 2018a)). *Abbreviations:* AF, assessment factor; CTD, chemical toxicity distribution; ecoTTC, ecotoxicological Threshold of Toxicological Concern, PNEC, predicted no-effect concentration; Suppl.w, supplemented with; TL, trophic level.

For the chemicals for which no experimental ecotoxicological value could be found, a threshold concentration value was derived with the EnviroTox online tool and used to fill the data gap (Fig. 1). In the case of missing PNEC (i.e., for the calculation of Eq. (1), Section 2.3), an ecoTTC value was used; whereas in the case of missing EC_50_ (i.e., for the calculation of the Toxic Unit, Eq. (2), Section 2.3), a CTD-TC value was used. A flowchart describing the calculation steps is shown in Fig. 2. Briefly, the tool calculate first a geometric mean for each chemical, species and test type (acute or chronic) combination, if there are several ecotoxicological studies available. If there is only one study, it is taken as such. A second geometric mean is then calculated for each chemical across species, for each trophic level and test type combination (i.e., acute invertebrate). A workflow facilitate selection of the relevant geometric mean for this chemical (i.e., usually the most sensitive trophic level) and to apply to this geometric mean the relevant AF. This is done for each chemical present in the data set, i.e., each chemical gives one data point of the PNEC distribution. The complete dataset used (all chemicals/all trophic levels) is given in Table S3.

The European setting has been used with the EnviroTox tool, i.e., the PNECs have been calculated with the AF recommended by ECHA (2008), with some modification. The tool identifies different types of PNEC according to the underlying dataset and the AF that has been used to derive the PNEC. The various PNEC types with the European setting are described in Table S4. The main modification is that in the EnviroTox tool, it is possible to derive a PNEC for a chemical for which only one acute data point on one trophic level would be available, by using an AF of 10,000, or based on two acute data points from two different trophic levels, by using an AF of 5000, which would not usually be possible in the context of Registration, Evaluation, Authorisation and Restriction of Chemicals (REACH), for which a full acute dataset (i.e., at least acute data on three trophic levels) is usually required. This possibility has been added in the tool in order to take full advantage of the complete dataset; however, it has the disadvantage to introduce the use of high AF and to increase the uncertainty surrounding the PNEC derivation. The EnviroTox tool also gives the user the possibility to select the PNEC type one wants to be included in the distribution. Restricting the underlying dataset to chemicals with larger datasets, including chronic values, allows for the use of lower AFs and therefore reduces the uncertainties in the single chemical PNEC derivation, which is particularly important in the context of MRA, in which the uncertainties linked to the risk assessment of each individual chemical present in the mixture will systematically add up. Therefore, different configurations have been used and compared in this work to derive an ecoTTC value: (ecoTTC-1) using a PNEC distribution based on PNEC types 15 to 19, in order to restrict the PNECs used to the ones strictly following the REACH recommendation; (ecoTTC-2) using a PNEC distribution of chemicals for which chronic toxicity value were also available, (i.e., PNEC type 17, 18, 19 only), for which the AFs applied are lower because the underlying dataset is more robust; (ecoTTC-3) using a PNEC distribution based on chemicals having a full chronic data set only (PNEC 19). Moreover, in configuration 1, 2, and 3, the PNEC distributions were also built for chemicals sub-grouped by MOA according to the Verhaar framework, to understand the influence that such grouping might have on the ecoTTC value. The Verhaar MOA framework has been chosen because it includes only four classes (non-polar narcotics, polar narcotics, non-specifically reactive and specifically reactive chemicals) without an in-depth description of specific mode of action. This framework was judged the most relevant, the aim being here to group chemicals according to their potential toxicity range (with toxicity of narcotics < non-specifically reactive chemicals < specifically reactive chemicals, generally).

The CTD-TC calculation process is shown in Fig. 2. It starts with the same first step as for the calculation of the ecoTTC, by calculating a geometric mean for each species and test type combination, if there are several studies available in the dataset. It is then possible to filter the data (i.e., if we want to build a distribution based on acute data only or chronic data only) and to split the analysis by species or trophic level. Then, a second geometric mean is calculated for each trophic level across species, for the selected species or trophic level. Each chemical will then represent one datapoint of the distribution.

The CTD-TC values have been calculated based on acute data only, split by trophic level, as the aim is to use this CTD-TC as a surrogate value for a missing LC_50_ for a given trophic level. CTD-TC values were derived 1) on the whole dataset, and 2) on chemicals sub-grouped by MOA. The whole process of the PNEC calculation and ecoTTC or CTD-TC derivation is explained in detail in the User Guide of the online tool (HESI, 2018a) and is freely available on the EnviroTox website (https://envirotoxdatabase.org).

### 2.3 MRA calculation methods

For this case study the framework proposed by Backhaus and Faust (2012) has been applied, which uses the sum of risk quotients as a surrogate for CA-based predictions. The framework is made of two calculation steps: the first step consists in the calculation of the risk quotient for the mixture (RQ_mix_), based on the summation of the risk quotients of the individual substances (RQ_i_) in which the RQ_i_ is defined as the measured exposure concentration (MEC; concentration of chemical i analyzed in a water sample) divided by the PNEC (Eq. (1)):

(1) $${\mathrm{RQ}}_{\mathrm{mix}}=\sum_{i=1}^{n}{\mathrm{RQ}}_{i}=\sum_{i=1}^{n}\frac{{\mathrm{MEC}}_{i}}{{\mathrm{PNEC}}_{i}}\;\text{with}\;{\mathrm{RQ}}_{i}=\frac{{\mathrm{MEC}}_{i}}{{\mathrm{PNEC}}_{i}}\;\text{and}\;{\mathrm{PNEC}}_{i}=\frac{\min\left(\mathrm{EC}50\;\mathrm{or NOEC fish},\mathrm{invert},\mathrm{algae}\right)i}{\mathrm{AF}}$$

This approach is conceptually different from CA because the involved PNECs might be based on different (groups of) species and using different AFs; however, summation of MEC/PNEC ratios can be used as a screening-level approach (Backhaus and Faust, 2012).

If a risk is identified (i.e., RQ_mix_ > 1), the MRA can be refined in the second calculation step, based on the sum of toxic units (STU) (Eq. (2)), applied for each trophic level:

(2) $${\mathrm{RQ}}_{\mathrm{STU}}=\max\left({\mathrm{STU}}_{\mathrm{algae},\;}\;{\mathrm{STU}}_{\mathrm{invert},\;}{\mathrm{STU}}_{\mathrm{fish}}\right)\times \mathrm{AF}=\max\left(\sum_{i=1}^{n}\frac{\mathrm{MECi}}{\mathrm{EC}50\;i,\mathrm{algae}},\sum_{i=1}^{n}\frac{\mathrm{MECi}}{\mathrm{EC}50\;i,\mathrm{invert}},\sum_{i=1}^{n}\frac{\mathrm{MECi}}{\mathrm{EC}50\;i,\mathrm{fish}}\right)\times \mathrm{AF}$$

where RQ_STU_ is the risk quotient based on the STU for the mixtures.

In this approach, the toxic unit (TU) is defined as the ratio between the MEC and the EC_50_ for a given trophic level and a given chemical, and the risk quotient of the mixture is defined as the highest STU (summation of the TU of each chemical present in the mixture, by trophic level) between the three trophic levels (algae, fish, and daphnia) to which is applied an uncertainty factor of 1000. For further details, see Backhaus and Faust (2012) and Backhaus and Karlsson (2014).

For calculating the risk quotient of the mixture as defined in Eqs. (1), (2), we need both 1) exposure data, i.e., the concentration of the component in the mixture; in this work the monitoring concentration were used as stated in Section 2.1; 2) ecotoxicological reference values for each chemical present in the mixture, such as LC_50_ or the PNEC; in this work experimental data were used when available and threshold concentration were used when there was no experimental data (see Section 2.2).

## 3 Results and discussion

### 3.1 Calculation of the threshold concentrations

**Fig. 3:**
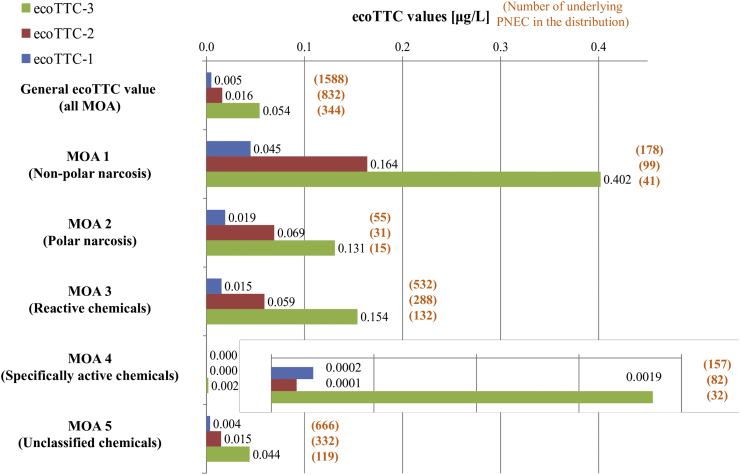
EcoTTC values, based on the whole dataset or by Verhaar MOA, in three configurations: ecoTTC-1: PNEC types 15 to 19; ecoTTC-2: PNEC type 17, 18, or 19 only; ecoTTC-3: PNEC type 19 only. n is the number of underlying PNEC values in the distribution. *Abbreviations:* ecoTTC, ecotoxicological Threshold of Toxicological Concern; MOA, mode of action; PNEC, predicted no-effect concentration.

The ecoTTC values derived for the three different configurations, with and without considering the MOA of the chemical, are given in Fig. 3 and Table S5. In addition, the number of underlying PNECs (i.e., number of chemicals, as one PNEC is calculated for each chemical) in each distribution is also reported in Fig. 3 and Table S5. The 95th confidence interval of each ecoTTC value is also given in Table S5.

The ecoTTC values derived for Verhaar MOA1 (non-polar narcotics), MOA2 (polar narcotics), and MOA3 (reactive chemicals) are of the same order of magnitude (respectively 4.5 10^−2^, 1.91 10^−2^, and 1.54 10^−2^ μg/L for ecoTTC-1), slightly higher than the ecoTTC value derived based on all MOA (4.98 10^−3^ μg/L), whereas the ecoTTC value derived for MOA4 (specifically acting chemicals) is much lower (2.04 10^−4^ μg/L). This suggests that the use of a general ecoTTC value might not be conservative enough for specifically acting chemicals (MOA4). This general trend is kept when considering ecoTTC-2 and ecoTTC-3.

If the PNEC distribution is based on chemicals for which chronic data are available (PNEC type 17, 18, or 19, i.e., ecoTTC-2), the AFs applied on the toxicological value used to derive the PNEC are lowered (10 to 100 instead of a factor of 1000 minimum when the PNEC is derived from acute data only) (ECHA, 2008). Given the size of the underlying database, even when restricting the dataset to the chemicals for which chronic data are available, it might still be possible to derive an ecoTTC value, and comparing the ecoTTC-1, ecoTTC-2 and ecoTTC-3 value therefore allows to assess the influence that might have the use of high AF in the PNEC distribution. The change from ecoTTC-1 to ecoTTC-2 translates into an increase of the general ecoTTC value (considering all MOA) from 4.98 10^−3^ to 1.61 10^−2^ μg/L, which corresponds to a factor 3.2. When looking at the MOA subgroups, this shift is maintained for MOA1 (non-polar narcotics), MOA2 (polar narcotics), and MOA3 (reactive chemicals) chemicals. For MOA4 (specifically acting) chemicals, the ecoTTC value derived based on PNEC distribution is nearly the same in configuration ecoTTC-1 and ecoTTC-2, which might be due to the structure of the underlying dataset: MOA4 chemicals are mostly data-rich chemicals for which chronic values are often available, therefore there will be less MOA4 chemicals with acute data only and PNEC type 15 or 16 might have less weight in the PNEC distribution.

When comparing ecoTTC-1 to ecoTTC-3, based on chemicals with a complete chronic dataset (i.e., chronic data for three different trophic levels and use of a factor 10 to derive the PNEC), the shift is even more pronounced: the ecoTTC value changes from 4.98 10^−3^ μg/L to 5.42 10^−2^, which corresponds to a factor of 10.88 (Table S5), for the general ecoTTC value (based on all MOA). However, the underlying dataset decreases from 1588 to 344 chemicals (all MOA included). When looking by MOA, this factor ranges from 6.9 for polar narcotics to 11.8 for MOA5 (unclassified chemicals), and the underlying datasets can become as small as 15 chemicals (polar narcotics). Therefore, it should also be checked that the increase in the ecoTTC value is mainly due to the decreased uncertainty and not the decrease in the data set (i.e., the exclusion of potentially more toxic chemicals from the dataset), and that the underlying data set is still considered representative to derive a meaningful threshold concentration. Another consequence of the decrease of the underlying dataset is an increase of the 95% confidence interval of the ecoTTC value (Table S5). More particularly, the upper confidence limits of the ecoTTC-3 value increase for all MOA, and to a lesser extend the upper limit of the ecoTTC-2 value for MOA 1 and MOA 2 chemicals. However, the lower confidence limit (the most relevant in this case) is less impacted.

### 3.2 Comparison of the ecoTTC values with PNEC values based on chemical-specific experimental data

**Fig. 4:**
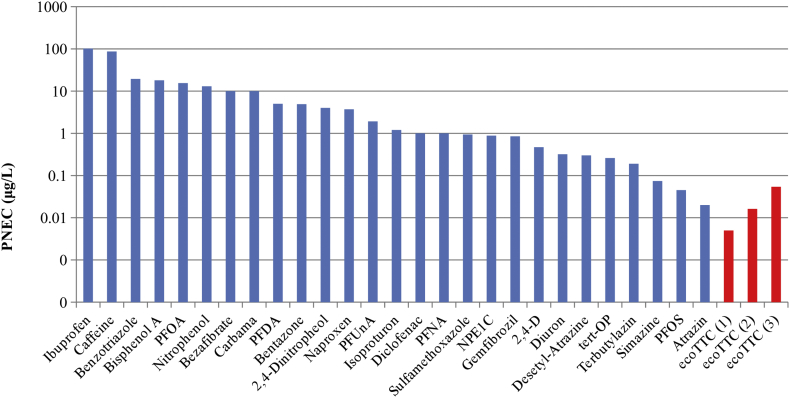
Experimental PNECs (blue bars) and general ecoTTC value (for all MOAs) (red bars). *Abbreviations:* ecoTTC, ecotoxicological Threshold of Toxicological Concern; MOA, mode of action; PNEC, predicted no-effect concentration. (For interpretation of the references to colour in this figure legend, the reader is referred to the web version of this article.)

The PNEC calculation details and the underlying ecotoxicological data are available in Table S2. Chemical-specific PNEC values are presented in Fig. 4, along with the general ecoTTC-1, ecoTTC-2, and ecoTTC-3 values (based on all MOA).

Fig. 4 shows that all the experimental PNEC values are higher than the ecoTTC-1 and ecoTTC-2 values based on all MOA; i.e., these ecoTTC values are conservative enough to cover all the PNECs in the exposure scenarios under investigation in this study. This is not the case for the ecoTTC-3 value, which suggests that the use of chemicals with a complete chronic dataset only to derive the ecoTTC value is probably too restrictive.

### 3.3 Calculation of the CTD-TC

The CTD-TCs calculated by MOA and by trophic level are given in Table 1. When considering all trophic levels, the same trend than for the ecoTTC value can be highlighted: the CTD-TC values for MOA1, MOA2, and MOA3 chemicals are quite similar, while the CTD-TC value for MOA4 is around two orders of magnitude below. Looking at trophic levels (and considering all MOA), overall, invertebrates seem to be the most sensitive with the lowest CTD-TC value (14.7 μg/L), closely followed by algae (19.9 μg/L). Fish seem to be less sensitive with a CTD-TC of 49.5 μg/L. Regarding the analysis by MOA, the trend is the same for MOA1 chemicals. For MOA2 and MOA3 chemicals, algae are the most sensitive (with a CTD-TC of 78.5 and 40.5 μg/L respectively), with fish still being the least sensitive trophic level (CTD-TC of 913.4 and 126.5 μg/L respectively), invertebrate being in the middle (171.5 and 114.8 μg/L respectively). For specifically acting chemicals (MOA4), invertebrates are the most sensitive (0.3 μg/L) closely followed by fish (2.4 μg/L), which seems to be particularly sensitive to this type of chemicals, whereas algae became the least sensitive trophic level (60.4 μg/L). This could be because the Verhaar MOA framework is based on fish toxicity, i.e., they are particularly less sensitive to MOA1 and MOA2 chemicals, but particularly sensitive to MOA4 chemicals.

**Table 1:** CTD-TC values based on acute data only.

CTD-TC^a^ (μg/L)	All chemicals/MOA (n)	MOA 1 (n)	MOA 2 (n)	MOA 3 (n)	MOA 4 (n)	MOA 5 (n)
All trophic levels	35.6 (3691)	155.5 (441)	385.7 (105)	119.3 (1243)	2.2 (319)	23.0 (1583)
*Ratio*^b^ *CTD-TC/ecoTTC-2*	*2211*	*948*	*5581*	*2019*	*17,000*	*1543*
Algae	19.9 (1216)	94.3 (138)	78.5 (54)	40.5 (449)	60.4 (99)	5.1 (477)
Fish	49.5 (2817)	314.8 (341)	913.4 (86)	126.5 (939)	2.4 (282)	44.5 (1170)
Invertebrates	14.7 (2307)	81.8 (264)	171.5 (76)	114.8 (732)	0.3 (245)	10.1 (990)

*Abbreviations:* CTD, chemical toxicity distribution; ecoTTC, ecotoxicological Threshold of Toxicological Concern; MOA, mode of action.

The line in italic provides the ratio between two threshold concentrations discussed in the text, the CTD-TC and the ecoTTC-2; the italic aims at differentiating this line to the other.

a CTD-TC values are presented based on the whole dataset, or stratified by MOA (column) and trophic level (line). n is the number of chemicals in the distribution.

b The ratio has been calculated based on the ecoTTC-2 value. It has to be noted that the CTD-TC does not use an assessment factor so that it is expected to be orders of magnitude higher than the ecoTTC.

There is a factor of around 10^3^ when comparing the CTD-TCs (including all trophic level) based on acute data only, to the corresponding ecoTTC-2 (all MOA or by MOA) (Table 1); respectively a factor of 2211 including all MOA, 948 for MOA1 chemicals, 5581 for MOA2 chemicals, and 2019 for MOA3 chemicals. This reflects the fact that PNEC distributions used in this context integrate chronic data (i.e., which values are usually lower than acute data) and additional uncertainties factors. For the MOA4 chemicals, this factor goes up to 17,000, which shows that the ecoTTC value for this group of chemicals is particularly low and conservative when compared to the CTD-TC value.

### 3.4 MRA

The slight difference obtained between the ecoTTC value for MOA 1, MOA 2 and MOA 3 chemicals (Table S5 and Fig. 3) suggest that differentiating those MOA1, MOA2, and MOA3 chemicals will not have a substantial impact on the resulting RQ_mix_ when using this exposure dataset, but when the use of a general ecoTTC value (i.e., based on all MOA) leads to a RQ_mix_ slightly above 1, the refinement of the RQ_mix_ by MOA could be necessary to investigate if there is a concern.

The mixture risk quotient (RQ_mix_) has been calculated according to Eq. (1), using the experimental PNEC values for the chemicals for which experimental data were available, and the ecoTTC-2 value for data gap filling. EcoTTC-2 was chosen in order to decrease the uncertainty factors included in the PNEC calculations, though still allowing a satisfactory conservatism. This calculation has been done: 1) with the general ecoTTC-2 value (no MOA consideration) for the chemicals for which no experimental data were available (i.e., perfluorohexanoate (PFHxA), perfluoroheptanoate (PFHpA), perfluoroundecanoate (PFUnA), desetylterbutylazine, methylbenzotriazole; none of those were profiled as specifically acting), and 2) using the ecoTTC-2 value considering the MOA of the chemical.

The detailed calculations are presented in Table S6, with the individual RQi values for each chemical. The resulting RQ_mix_ is given in Table 2, along with the number of chemicals for which an ecoTTC-2 value has been used in the calculation of the RQ_mix_, the number of chemicals detected in the scenarios, the two individual chemical RQ_i_ driving the RQ_mix_, and their corresponding percentage of the RQ_mix_.

**Table 2:**
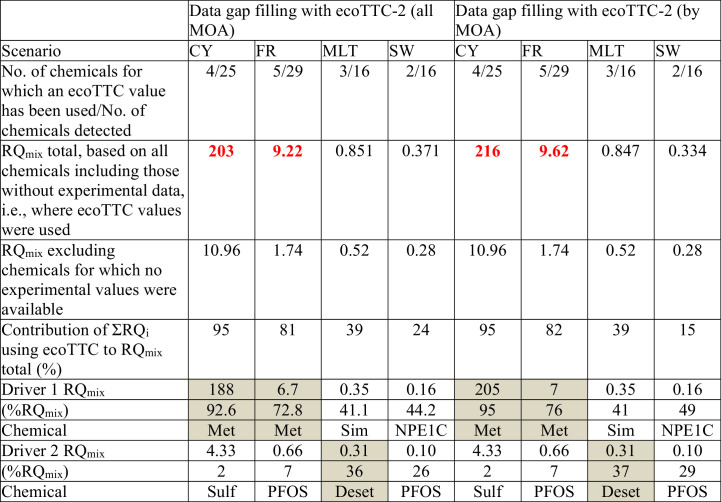
Risk quotients for the combined exposure to the analyzed chemicals (RQ_mix_) calculated for each scenario, using experimental PNEC values and when no experimental data were available, ecoTTC-2 value; based on general ecoTTC-2 including all MOA (left) and by MOA (right).

RQ_mix_ > 1 indicates a potential risk (bold). The cells in gray indicate that the corresponding RQ_i_ is derived using an ecoTTC-2 value. ∑RQ_i_ with ecoTTC value: Sum of the RQ_i_ based on ecoTTC value, and their % of the RQ_mix_ total. Driver RQi 1 and 2: the two chemicals with the highest RQ_i_ in the mixture. *Abbreviations:* CY, Cyprus; Deset, desethylterbutylazine; FR, France; Met, methylbenzotriazole; MLT, Malta; PFOA, perfluorooctanoic acid; PFOS, perfluorooctanesulfonic acid; Sim, simazine; SW, Sweden; Sulf, sulfamethoxazole.

In the four site-specific scenarios, the number of chemicals for which an ecoTTC value has been used for data gap filling to derive the RQ_i_ ranges from to 2 (SW) to 5 (FR). The RQ_mix_ values range from 0.371 to 203. Of the four scenarios studied, two result in a RQ_mix_ < 1, thus indicating low concern and no need for refinement (SW and MLT, with respectively a RQ_mix_ of 0.371 and 0.851). The other two scenarios have RQ_mix_ that ranges from 9 to 203. The RQ_mix_ is in both sites heavily driven by the RQ_i_ values calculated using ecoTTC values, which account for 81 and 95% of the RQ_mix_ total, respectively. The high impact of the ecoTTC values on the predicted overall mixture risk is not surprising, as the ecoTTC values have to be low enough to cover most of chemicals' toxicities.

In the CY and FR scenario, the chemical driving the overall RQ_mix_ is methylbenzotriazole, for which RQ_i_ is based on the use of the ecoTTC value, and which accounts for 92.6 and 72.8% of the RQ_mix_ total, respectively. The second main drivers of the RQ_mix_ for those two scenarios are sulfamethoxazole and PFOS, which account for respectively 2 and 7% of the RQ_mix_. Their RQ_i_ have been calculated based on experimental PNECs. For the MLT and SW scenario, the first ranking drivers are Simazine and NPE1C, respectively, which account for 41 and 44% of the RQ_mix_ total, and for which the RQ_i_ have been calculated on experimental PNECs. The second main drivers for those two scenarios are desethylterbutylazine and PFOS, which account for 36 and 26% of the RQ_mix_, respectively.

When using the MOA-tailored ecoTTC values, the RQ_mix_ total ranges from 0.334 to 216; i.e., two scenarios have a slightly decreased RQ_mix_ (MLT and SW for within the RQ_mix_ respectively decrease from 0.851 to 0.847 and from 0.371 to 0.334), whereas the other two have a RQ_mix_ slightly increased (CY and FR for which the RQ_mix_ respectively change from 203 to 216 and from 9.22 to 9.62) but the identification of concern is not modified. The ∑RQ_i_ calculated with the ecoTTC value account for 15 to 95% of the RQ_mix_ total, depending on the scenarios. The chemicals driving the RQ_mix_ remain the same. Therefore, in this context, the use of the MOA does not help in refining; however, it should be taken into consideration that in this case, three of the chemicals for which the ecoTTC value has been used are classified MOA5 and two are classified MOA3, i.e., their respective ecoTTC value is quite similar to the general one. For specifically acting chemicals (MOA4) the ecoTTC value is much lower, as shown in Table S5, and for narcotic chemicals (MOA1) the ecoTTC is higher and could help in refining to some extent.

To give an idea of how the MOA could influence the RQ_mix_, the “allowed” chemical burden for MOA1 and MOA4 chemicals not exceeding the RQ_mix_ = 1 have been compared. In this case, the overall concentration of chemicals in a river as the sum of MECs would equal the PNEC, i.e., the ecoTTC value according to Eq. (1). Therefore, an environmental concentration of 164 ng/L of a mixture of narcotic chemicals would be allowed before triggering a refinement, whereas this concentration would go down to 0.123 ng/L for specifically acting chemicals. If no MOA considerations are included an overall chemical burden of 16 ng/L would be the threshold of concern. Obviously, these choices have a large impact, and comparing the ecoTTC values to the current concentrations frequently found in the aquatic environment, as in the monitoring dataset used here (Loos et al., 2009), would trigger a concern in most cases if only ecoTTC would be considered for all compounds.

### 3.5 Refinement by trophic level

The MRA has been refined by trophic level for the two scenarios FR and CY where a need for refinement was identified. For this purpose, CTD-TC values are used instead of ecoTTC values. Results are presented in Table 3 and the detailed calculations are given in Table S7.

**Table 3:**
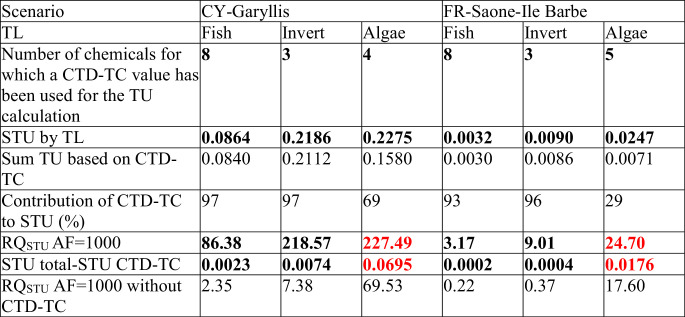
Calculation of the STU and related mixture risk quotient RQ_STU_ (using the STU multiplied by an assessment factor of 1000) for each scenario, by trophic level.

The highest RQ_STU_ is highlighted in red. *Abbreviations:* AF, assessment factor; CTD-TC, chemical toxicity distribution-threshold concentration; CY, Cyprus; Deset, desethylterbutylazine; FR, France; Met, methylbenzotriazole; MLT, Malta; Sim, simazine; STU, sum of toxic units; SW, Sweden; Sulf, sulfamethoxazole; TL, trophic level; TU, toxic unit.

The refinement by trophic level shows still a risk (and therefore a need for further refinement) for both scenarios and all trophic levels (RQ_STU_ > 1). This can be explained by the fact that in most of the cases, a surrogate CTD-TC was used for more chemicals than in step 1, to fill the data gap for chemicals for which an acute ecotoxicity value was missing for a given trophic level. As an example, in both scenarios up to eight chemicals were missing acute LC_50_ values for fish, and three chemicals for invertebrates. For algae, four and five chemicals were missing acute toxicity data in scenario CY and FR, respectively. It is worth noting that algae have the highest RQ_STU_ for both scenarios, even if the contribution of filling data gaps using CTD-TC values has the lowest impact for algae. The lowest RQ_STU_ was found for fish. As a risk was identified for all trophic levels, a further refinement would be needed, such as using quantitative structure–activity relationship (QSAR) data for acute aquatic toxicity in step 2, to move further. If we consider the RQ_STU_ without the CTD-TC value, a risk would have still been identified in both scenarios, as the trophic level with the highest RQ_STU_ should be used to calculate the final RQ_STU_ for the mixture. However, in the FR scenario, the calculated RQ_STU_ for invertebrates and fish is then below 1, although a CTD-TC has been used for three and eight chemicals, respectively. It has to be noted that this study was not performed to finally assess potential risks based on the chemicals detected at the represented river sites, but only to illustrate the potential use of ecoTTC and CTD-TC values for data gap filling.

EcoTTC values calculated using the EnviroTox database and tool can be used for data gap filling in MRA, but due to the inherent conservatism of the approach, its use in practice can only be limited to some chemicals in the mixture. If the ecoTTC values are used for more chemicals, the RQmix will systematically exceed the threshold of 1 triggering the need for a refinement. The RQ_i_ calculated with an ecoTTC value to fill data gaps in the MRA usually drives the final RQ_mix_, as this surrogate value is generally substantially lower than individual PNECs based on experimental data. The ecoTTC values are based on distributions of PNECs covering many different organisms and chemicals; applying the standard regulatory safety factors as done in single substance risk assessment to derive the PNEC and calculating the fifth percentile of the distribution curve makes the derived threshold concentration very conservative. For instance, if we compare the ecoTTC value (based on PNEC distribution, i.e. including AF) and the corresponding CTD-TC value (based on e.g. LC50 without AF), the factor between them is around 1000 and depends on the MOA (Table 1), which is due to the fact that most of the chemicals in the dataset have only acute data, to which a factor of 1000 will be applied for the PNEC derivation. In other words, we should apply a factor of at least 1000 to have a CTD-TC value within the same range as the ecoTTC value. This factor 1000 is far away from the factor of 1 to 5 used to derive a PNEC from the fifth percentile of a species sensitivity distribution (SSD – distribution of ecotoxicological data without the prior application of an AF) in the context of REACH (ECHA, 2008). However, it has to be highlighted that an SSD is built using the data from one chemical only, whereas in our case the statistical distributions included a wide number of chemicals.

Nevertheless, this conservatism is preferred in a screening step. Besides, the EnviroTox tool allows to select the type of PNEC to include in the distribution, which allows to some extent to decrease the uncertainties' linked to the use of PNEC and their related AF by selecting a more robust dataset. On the other hand, one should keep in mind when using a threshold value based on the fifth percentile of a statistical distribution for several chemicals that there is still a 5% risk for each of those chemicals that it has a real toxicity below this threshold concentration (Backhaus, 2014).

This case study focused on polar organic chemicals only based on the underlying monitoring data set. However, metals are also major environmental toxicants, and could be addressed if needed, as the EnviroTox database include >17 000 data records on 317 chemicals flagged as metals, across 747 species. Thus, threshold concentrations could be derived for this type of toxicant if needed. However it has to be kept in mind that metal toxicity can be significantly impacted by test conditions (e.g., water hardness), and because the database does not collect test condition information, study results can be difficult to interpret. Moreover, metal toxicity often displays hormesis, where the toxicity response curve will be j or u-shaped, therefore test design and the spacing of doses can alter the interpretation. Thus metals toxicity data should be used with caution.

In addition, the difficulties inherent to MRA also hold true for this approach. As an example, even if the use of MOA can help refine the risk assessment in some cases, the frequent lack of mechanistic knowledge of chemical toxicity still hinders regulatory ecotoxicology, especially given the complexity of the task when considering several species. This screening approach also does not consider potential interaction. However the use of both the assessment factor in the PNEC derivation and the 5th percentile of the statistical distribution make this approach conservative enough to cover the majority of the interactions reported in the literature at low exposure levels, which rarely exceed a 10-fold factor (Cedergreen, 2014). Moreover, the approach was not validated with toxicity data obtained in situ, as it was not possible to sample the site and the sampling nowadays would have been different from the monitoring data from 2014.

To conclude, this study showed that it was possible to use those threshold concentrations for missing toxicity values in MRA for one to three chemicals in the mixture. Therefore this approach could be used for data gap filling for data-poor chemicals, but not as a screening approach for all chemicals present in the mixtures, as this has been proposed for human health (Boobis et al., 2011). This is also true for the use of the CTD-TC value in the second step (use of the RQ_STU_ by trophic level), especially since there were usually more data gaps to be filled with a surrogate value than in step 1 (use of the RQ_mix_ based on PNEC). The consideration of the MOA might be helpful in some cases, i.e., narcotic chemicals or specifically acting chemicals.

## Appendix A Supplementary data

Supplementary tables Image 1
